# Prevalence and predictors of unintended pregnancy among antenatal women in Sierra Leone: A cross-sectional study

**DOI:** 10.1371/journal.pgph.0005086

**Published:** 2025-08-22

**Authors:** Alieu Kanu, Abdul Karim Bah, Michele Orsi, Iye Pateh Jalloh, Fatmata Yeanoh Turay, Sulaiman Kanu, Edgardo Somigliana, Fatima Jalloh, Michael Ezeanochie, Mohamed B. Jalloh

**Affiliations:** 1 Princess Christian Maternity Hospital, University of Sierra Leone Teaching Hospitals Complex, Freetown, Sierra Leone; 2 Fondazione IRCCS Ca’ Granda Ospedale Maggiore Policlinico, Milan, Italy; 3 College of Medicine and Allied Health Sciences, University of Sierra Leone, Freetown, Sierra Leone; 4 Department of Clinical Sciences and Community Health, University of Milan, Milan, Italy; 5 Department of Medicine, McMaster University, Hamilton, Ontario, Canada; VART Consulting PVT LTD, INDIA

## Abstract

Unintended pregnancy remains a major contributor to adverse maternal and child health outcomes in sub-Saharan Africa, yet recent facility-based data from Sierra Leone are scarce. We aimed to determine the prevalence and identify factors associated with unintended pregnancies among antenatal clinic attendees at a major tertiary maternity hospital in Sierra Leone. We surveyed 1005 first-visit antenatal attendees at Princess Christian Maternity Hospital in Freetown between 19 March and 30 June 2024, using systematic sampling and multivariable logistic regression to identify independent predictors. Overall, 31.8% of women (95% CI 29.0 – 34.7) reported the current pregnancy as unintended; most were mistimed (30.0%) and the remainder unwanted (1.8%). Higher odds of unintended pregnancy were observed among women younger than 20 years (aOR 3.57, 95% CI 2.30 – 5.55), those who were unmarried (aOR 3.73, 95% CI 2.60 – 5.36), and those who were unemployed or students (aOR 1.74, 95% CI 1.25 – 2.42). Open partner communication about pregnancy (aOR 0.10, 95% CI 0.07 – 0.16) and partner desire for the pregnancy (aOR 0.05, 95% CI 0.03 – 0.09) were strongly protective. Nearly one in three pregnancies at Sierra Leone’s principal referral maternity hospital is therefore unintended, with the burden falling on adolescents, unmarried women, and those with limited economic means. Interventions that integrate youth-friendly contraception services, partner-centred counselling, and broader female economic empowerment should be prioritised to reduce unintended pregnancies and improve maternal and child health.

## Introduction

Unintended pregnancy, defined as a conception that is either mistimed or unwanted at the time of occurrence, remains a major public-health concern with profound implications for maternal, neonatal, and child survival [1,2]. The most recent global synthesis estimates 121 million unintended pregnancies annually between 2015 and 2019, equivalent to 64 per 1000 women aged 15–49 years, and shows little progress since 2010 [3]. Unintended pregnancies contribute to nearly 61% of the 73 million induced abortions that take place each year, many of which are unsafe in low-resource settings [4].

Sub-Saharan Africa (SSA) bears a disproportionate share of this burden. A pooled analysis of 17, 000 survey respondents across 36 SSA countries reported a 30% prevalence of unintended pregnancy, with women who were young, unmarried, of high parity, or socio-economically disadvantaged at greatest risk [5]. Another systematic review found similarly high rates in West Africa and highlighted restrictive abortion laws, low modern-contraceptive coverage, and entrenched gender norms as persistent drivers [6].

Modern contraceptive uptake is still modest in the region. Only one in four sexually active women in SSA uses a modern method, despite robust evidence that effective contraception could avert two-thirds of unintended pregnancies in low-income countries [7].

Sierra Leone mirrors, and in some respects exceeds, these regional challenges. The country records one of the world’s highest maternal-mortality ratios (354 deaths per 100 000 live births) [8], and unsafe abortion accounts for an estimated 7% of those deaths [9]. According to the 2019 Sierra Leone Demographic and Health Survey (SLDHS), 17% of recent pregnancies were unintended; 14% were mistimed and 3% unwanted [10]. Modern-contraceptive prevalence improved from 20% in 2013 to 24% in 2019, yet remains far below the level needed to curb unintended pregnancy meaningfully [10]. Although self-reports in the 2013 and 2019 Sierra Leone Demographic and Health Surveys hover around 9 percent of women ever having had an induced abortion, more robust national estimates using the Abortion Incidence Complications Method put the annual rate far higher, about 44.2 abortions per 1000 women aged 15–49 in 2021 [11].

Beyond the epidemiological burden, unintended pregnancy in Sierra Leone has distinctive social ramifications. Qualitative work shows that women, particularly adolescents and unmarried women, perceive unsafe abortion as a lesser threat than the severe social sanctions attached to non-marital child-bearing, interrupted education, and loss of economic opportunities [12].

National household surveys provide invaluable population-level estimates, but they cannot characterise the clinical sequelae of unintended pregnancy, nor can they describe risk profiles of women who present for hospital-based antenatal care. Princess Christian Maternity Hospital (PCMH) in Freetown is Sierra Leone’s largest maternity referral centre, responsible for high-risk deliveries from across the country. Yet no published study has quantified the prevalence of unintended pregnancy among its antenatal clientele, or examined how socio-demographic, reproductive, and health-system factors intersect with pregnancy intention and perinatal outcomes in this high-acuity setting. Accordingly, evidence to inform facility-level quality-improvement and national policy remains limited.

To address this gap, our study aimed to comprehensively investigate the prevalence, determinants, and health implications of unintended pregnancy among women attending antenatal care at a major tertiary referral maternity hospital in Sierra Leone. Findings could inform targeted interventions to reduce unintended pregnancy and its complications in Sierra Leone and contribute to the broader West-African evidence base.

## Methods

### Study design and setting

We conducted a cross‐sectional, questionnaire‐based study at PCMH, a public tertiary facility affiliated with the University of Sierra Leone Teaching Hospitals Complex in Freetown, Sierra Leone. PCMH functions both as a primary provider of antenatal care for uncomplicated cases and as a referral center for complex cases from smaller local facilities. First antenatal care (ANC) bookings are managed by medical doctors, and the hospital facilitates approximately 7,000–8,000 deliveries annually, 30–40% of which are referrals for high‐risk conditions or obstetric complications.

### Participants and sampling

Eligible participants were women of reproductive age attending their first ANC visit between 19 March and 30 June 2024. We excluded women who were too ill to participate or who had diagnosed psychiatric disorders or cognitive impairments that could compromise informed consent or accurate responses. Using systematic sampling, every third eligible woman after triage was invited to participate until the target sample of 1005 was reached. Although systematic sampling can introduce bias if patient flow is non-random, strict adherence to the recruitment interval helped mitigate this risk.

### Data collection

Data were obtained via a semi-structured questionnaire administered in a dedicated private area within the antenatal clinic by trained research assistants. The questionnaire was developed based on a comprehensive review of previous studies [5,13–15] and expert consultations to ensure contextual relevance and content validity. To refine the instrument, pilot testing was conducted with 10 community representatives. We employed a culturally sensitive translation process, including forward and backward translation of all participant-facing materials, to ensure language accuracy and clarity. Feedback from the pilot participants confirmed the comprehensibility and cultural appropriateness of the materials. Research assistants received thorough training in standardized interview techniques and ethical procedures to ensure consistency and data integrity.

### Study variables

#### Dependent variable.

Pregnancy intention was the primary outcome, classified as intended or unintended. An intended pregnancy occurred when desired, whereas an unintended pregnancy was either mistimed (earlier than desired) or unwanted.

#### Independent variables.

Independent variables encompassed a range of sociodemographic and partner-related factors:

- **Demographic and socioeconomic variables:**
  - Age (<20, ≥20 years)
  - Religion (Islam, Christianity, Others)
  - Parity: defined as the number of previous live births and grouped as
    - Nulliparous (parity 0)
    - Primiparous (parity 1)
    - Multiparous (parity ≥2)
  - Employment status (merchant/self-employed, government employed, housewife, unemployed/student)
  - Education (non-formal, primary, secondary, tertiary)
  - Marital status (never married, married, divorced/separated/widowed, cohabiting†)
- **Partner and reproductive health variables:**
  - Any prior use of modern contraceptive (yes/no)
  - Communication with partner about pregnancy (yes/no)
  - Communication with partner about contraception (yes/no)
  - Partner’s desire for pregnancy (yes/no)
  - Occurrence of pregnancy because of non-consensual sexual intercourse (yes/no)
  - Willingness to receive family planning education (yes/no)
- *Cohabiting* denotes partners living together in a shared household without legal marriage.
- Notes on other labels in the tables
  - Others: refers to religious affiliations that do not fall under the major categories in Sierra Leone listed—Islam and Christianity.

### Statistical analysis

Continuous variables were first assessed for normality using the Kolmogorov-Smirnov test. Normally distributed data are presented as means with standard deviations, whereas non-normally distributed variables are summarized as medians with interquartile ranges. Categorical variables were analyzed using chi-square or Fisher’s exact tests, as appropriate. Multivariable binary logistic regression models were employed to evaluate the associations between unintended pregnancy and associated factors, with adjusted odds ratios (aOR) and 95% confidence intervals (CI) reported. To comprehensively investigate the diverse determinants of unexpected pregnancy, our multivariable binary logistic regression analysis was conducted in two conceptually distinct stages. Model 1 primarily assessed the contribution of maternal sociodemographic factors. Subsequently, Model 2 examined family planning and partner-related factors, with adjustment for those maternal sociodemographic variables that demonstrated statistical significance in Model 1. This hierarchical approach was adopted to enhance the interpretability of distinct risk factor domains and to facilitate the development of targeted public health interventions. Multicollinearity among independent variables in the multivariable logistic regression model was assessed using Variance Inflation Factors (VIF) and pairwise correlation coefficients. Variables exhibiting VIF values below 5, specifically ‘Age < 20 years’ (VIF = 2.5) and ‘Nulliparity’ (VIF = 2.3) despite a moderate pairwise correlation (r = 0.55), were retained in the final model due to their established clinical relevance, distinct conceptual contributions to the prediction of unexpected pregnancy and potential implications for policy makers. Considering rigorous training of data collectors and the provision of a dedicated, private area for interviews within the antenatal care setting, missing data were minimal, constituting no more than 2.0% for any single variable. Following accurate review of the dataset, we assumed that missingness occurred randomly and was not systematically related to specific participant characteristics or variable values. Consequently, listwise deletion was applied for all regression models, ensuring analyses were based on complete cases. Statistical significance was set at a p-value < 0.05, and all analyses were performed using SPSS version 27 (IBM Corp., Armonk, NY, USA).

### Participant and public involvement statement

Due to unexpected delays and limited time, we could not engage participants or the public in the design, implementation, or reporting stages of the study. Nevertheless, we are now planning to enhance public and stakeholder involvement when disseminating our research results.

### Ethical considerations

This study received approval from the Sierra Leone Ethics and Scientific Review Committee (SLESRC No: 005/03/2024). The SLESRC, operating under the Ministry of Health’s Directorate of Training and Research, is responsible for the ethical and scientific review of research protocols in Sierra Leone. It ensures that studies involving human participants adhere to ethical principles and established guidelines. Prior to participation, we obtained written informed consent from all individuals. Participants were assured of confidentiality, anonymity, and their right to withdraw at any time without consequences. To safeguard their data, we implemented strict protection measures, including de-identification of personal information and secure data storage.

## Results

### Sociodemographic characteristics of participants (n=1,005)

Table 1 presents the sociodemographic characteristics of 1,005 pregnant women who attended the antenatal clinic at PCMH during the study period. The majority of participants (88.8%) were 20 years of age or older. The mean (standard deviation) age was 25.7 (5.37) years. Islam was the predominant faith, with 77.6% of participants identifying as Muslim. Most women (42.0%) were multiparous, (37.0) were primiparous, while 20.9% were nulliparous. Nearly half (48.2%) of the respondents were self-employed, 20.1% were housewives, and 26.4% were either unemployed or students. The distribution was diverse, with 57.4% having completed secondary education, 17.4% reaching the tertiary level, and 20.6% reporting no formal education. The majority (73.0%) were married, while 18.0% were single. A substantial proportion (71.0%) of respondents reported previous use of modern contraceptives, whereas 29.0% had never used them.

**Table 1:** Socio-demographic characteristics of 1005 participants.

Variable	Number of respondents n and percent (%);
Age (Years)	
<20	113 (11.2)
≥20	892 (88.8)
*Mean age (standard deviation)*	25.7 (5.37)
***Religion***	
Islam	771 (76.7)
Christianity	232 (23.1)
Others	2 (0.2)
**Parity**	
0	210 (20.9)
1	372 (37.0)
≥2	423 (42.1)
**Employment**	
Merchant/ self-employed	484 (48.2)
Government employed	53 (5.3)
Housewife	202 (20.1)
Unemployed/ student	266 (26.4)
**Education**	
Non-formal	207 (20.6)
Primary	45 (4.5)
Secondary	578 (57.5)
Tertiary	175 (17.4)
**Marital Status**	
Never married	181 (18.0)
Married	734 (73.0)
Divorced/Separated/ widowed	4 (0.4)
Cohabiting	86 (8.6)
**Modern Contraceptive Use**	
No	291 (29.0)
Yes	714 (71.0)
**Intended versus Unintended Pregnancy**	
Intended pregnancy	685 (68.2)
Unintended pregnancy	320 (31.8)
* Mistimed*	302 (30.0)
* Unwanted*	18 (1.8)

Regarding pregnancy intention, 320 participants (31.8%) reported unintended pregnancies. Of these, 302 (30.0%) were classified as mistimed, and 18 (1.8%) as unwanted. The remaining 685 participants (68.2%) reported their pregnancies as intended.

As shown in Table 2, women under 20 years of age had a significantly higher prevalence of unintended pregnancy compared to older women (61.1% vs. 28.1%, p < 0.001). Nulliparous women also reported higher rates of unintended pregnancy than multiparous women (39.1% vs. 29.6-30.3%, p = 0.012). Marital status was strongly associated with pregnancy intention, with never-married women reporting the highest prevalence of unintended pregnancies (59.7%), followed by divorced/separated/widowed women (50.0%), while married women had the lowest prevalence (24.3%) (p < 0.001).

**Table 2:** Pregnancy intentions by maternal characteristics (n. = 1005).

Characteristics	Unintended n = 320 (%)	Intended n = 685 (%)	P value
***Age (years)***			
<20	69 (61.1)	44 (38.9)	***<0.001***
≥20	251 (28.1)	641 (71.9)	
***Religion***			
Islam	250 (32.4)	521 (67.6)	***0.470***
Christianity	69 (29.7)	163 (70.3)	
Others	1 (50.0)	1 (50.0)	
***Parity***			
0	82 (39.1)	128 (60.9)	***0.012***
1	110 (29.6)	262 (70.4)	
≥2	128 (30.3)	295 (69.7)	
**Employment**			
Merchant/self-employed	145 (30.0)	339 (70.0)	
Government employed	8 (15.1)	45 (84.9)	
Housewife	46 (22.8)	156 (77.2)	0.002
Unemployed/ Student	121 (45.5)	145 (54.5)	<0.001
***Education***			*0.260*
Non-formal	58 (28.0)	149 (72.0)	
Primary	8 (17.8)	37 (82.2)	
Secondary	204 (35.3)	374 (64.7)	
Tertiary	50 (28.6)	125 (71.4)	
***Marital status***			
Never Married	108 (59.7)	73 (40.3)	< 0.001
Married	179 (24.3)	555 (75.6)	
Divorced/Separated/ widowed	2 (50.0)	2 (50.0)	
Cohabiting	31 (36.0)	55 (64.0)	
***Modern contraceptive use***			
No	107 (36.8)	184 (63.2)	0.020
Yes	213 (29.8)	501 (70.2)	

### Sociodemographic characteristics independently associated with unintended pregnancy

Multivariate analysis revealed that maternal age below 20 years (aOR: 3.57, 95% CI: 2.30-5.55, p < 0.001), non-married status (aOR: 3.73, 95% CI: 2.60-5.36, p < 0.001), and being unemployed or a student (aOR: 1.74, 95% CI: 1.25-2.42, p = 0.001) were independently associated with unintended pregnancy (Table 3).

**Table 3:** Logistic regression analysis – Model 1: sociodemographic characteristics independently associated with unintended pregnancy (n. = 1001).

Characteristics	Crude OR (95% CI)	P value	aOR (95% CI)	P value
Age < 20	4.01 [2.67 - 6.01]	< 0.001	3.57 [2.30 - 5.55]	< 0.001
Not married	4.27 [3.05 - 5.97]	< 0.001	3.73 [2.60 - 5.36]	< 0.001
Nulliparous	1.50 [1.09 - 2.06]	0.010	1.47 [1.00 - 2.17]	0.050
Unemployed or student	2.26 [1.69 - 3.03]	< 0.001	1.74 [1.25 - 2.42]	0.001

aOR, Adjusted odds ratio; CI, Confidence interval; OR, odds ratio.

Table 4 highlights partner-related factors. Women who communicated with their partner about pregnancy were significantly less likely to report an unintended pregnancy (aOR: 0.10, 95% CI: 0.07-0.16, p < 0.001). Similarly, women whose pregnancy was desired by their partner had lower odds of unintended pregnancy (aOR: 0.05, 95% CI: 0.03-0.09, p < 0.001). Also, women who reported to be victim of non-consensual sexual intercourse were more likely to report unintended pregnancy, although this association was not statistically significant.

**Table 4:** Logistic regression analysis – Model 2: family planning and partner related determinants of unintended pregnancy (n. = 998).

Characteristics	Unintended n = 320 (%)	Intended n = 685 (%)	P value	Crude OR (95% CI)	P value	aOR (95% CI)	P value
Communicated with partner about contraception	158 (49.4)	402 (58.7)	0.006	0.69 [0.53-0.90]	0.006	0.81 [0.60-1.07]	0.140
Communicated with partner about pregnancy	186 (58.1)	647 (94.5)	<0.001	0.08 [0.06-0.12]	<0.001	0.10 [0.07-0.16]	<0.001
Pregnancy desired by the partner	188 (58.8)	665 (97.1)	<0.001	0.04 [0.03-0.07]	<0.001	0.05 [0.03-0.09]	<0.001
Pregnancy as a result of non-consensual sexual intercourse	11 (3.4)	9 (1.3)	0.020	2.67 [1.10-6.52]	0.030	1.65 [0.61-4.49]	0.320
Willingness to accept education about family planning	303 (94.7)	602 (87.9)	<0.001	2.46 [1.43-4.22]	0.001	2.17[1.23-3.84]	0.007

aOR, Adjusted odds ratio; CI, Confidence interval; OR, odds ratio

The model was corrected for maternal age, marital status, parity and working status.

## Discussion

In this cross-sectional study conducted between March 19 and June 30, 2024, we assessed the prevalence and determinants of unintended pregnancies among women attending antenatal care at the primary referral maternity hospital (PCMH) in Freetown, Sierra Leone. We observed a prevalence of unintended pregnancy of 31.8%. The figure is almost double the 16.4% national average reported in the 2019 SLDHS [10], yet it closely mirrors the 33% prevalence documented for the Western Province where PCMH is situated [16]. The finding therefore reflects sub-national heterogeneity rather than an outlier effect of a tertiary facility. It also aligns with the pooled prevalence of 30.0% for SSA reported in a 36-country multilevel analysis, situating Sierra Leone within the regional pattern [5].

Adolescents were disproportionately affected. In the DHS, 38% of women aged 15–19 reported that their most recent pregnancy was unintended [16], a pattern we reproduced after multivariable adjustment. Consistent with regional literature, younger age (<20 years) was strongly associated with unintended pregnancies in our study, reflecting parallel findings from Ghana and Gambia [17,18] Potential explanations include limited sexual and reproductive health literacy among adolescents, economic dependence, and reduced agency in reproductive health decisions. Unmarried status remained strongly associated with unintended pregnancy, consistent with analyses from both Sierra Leone and Gambia that highlight compounded social and economic vulnerabilities for single young women [16,17]. Unemployment or student status also emerged as an independent predictor, echoing regional evidence that limited economic autonomy constrains access to modern contraception [5].

Nulliparity remained a significant predictor of unintended pregnancy even after accounting for its moderate collinearity with younger age, underscoring that the two characteristics represent distinct programmatic targets: first-time mothers need tailored contraceptive counselling, whereas adolescents benefit more from age-appropriate sexuality education. Retaining both variables in the model therefore guides more nuanced intervention design. Our observation aligns with findings from Kenya, where nulliparous women were more likely to experience unintended pregnancy [13], a pattern plausibly driven by limited knowledge of sexual and reproductive health.

Women who discussed pregnancy intentions with their partners and whose partners desired the pregnancy had lower odds of unintended pregnancy. Qualitative evidence from Sierra Leone reveals that factors such as partner denial of paternity, economic anxiety, and fear of social sanction often lead individuals to consider abortion or covert contraceptive use [12]. This suggests that couple-centered counseling could help mitigate reproductive coercion and enhance contraceptive uptake. Our findings are consistent with a broader body of evidence that supports partner engagement in family planning across LMICs [19–21]. Additionally, the well-documented links between reproductive coercion, intimate partner violence, and increased risk of unintended pregnancy further highlight the crucial role of partner dynamics [19,22].

### Programmatic and policy implications

Sierra Leone’s draft Safe Motherhood and Reproductive Health Bill [23] offers an opportunity to embed comprehensive sexuality education in national curricula, mandate youth-friendly corners in primary facilities and strengthen supply chains for long-acting reversible contraception. Evidence from mobile outreach programmes in West Africa indicates that bringing services to schools and markets increases modern-method uptake among adolescents; pilot implementation in the Western Province could provide a scalable model. Integrating routine partner counselling into antenatal care would further address the interpersonal drivers identified above.

### Strengths and limitations

A large sample size and adjustment for key confounders enhance the robustness of our estimates. Moderate collinearity between age and parity was examined with variance-inflation diagnostics and found acceptable for simultaneous inclusion. Limitations include reliance on self-reported pregnancy intention, which is susceptible to social-desirability bias that may inflate the proportion classified as wanted. Sensitive variables such as non-consensual intercourse are likely under-reported for the same reason. As a single urban referral site, PCMH may not represent rural primary facilities where supply-side barriers differ; caution is needed when extrapolating to those settings.

## Conclusion

Unintended pregnancy remains a substantial public-health challenge in urban Sierra Leone, especially among adolescents, unmarried women and those with limited economic means. Effective mitigation will require a multisectoral package that combines rights-based sexuality education, equitable access to modern contraception, economic empowerment of young women and active engagement of male partners. Strengthening these pillars is essential for improving maternal health outcomes and advancing reproductive autonomy in Sierra Leone.

## Supporting information

S1 Checklist. (DOCX)
